# Omega 3 fatty acid docosahexaenoic acid (DHA) mitigates inflammatory responses in experimental sepsis

**DOI:** 10.3389/fphar.2025.1708348

**Published:** 2025-11-28

**Authors:** Bianca Portugal Tavares de Moraes, Isabelle Moraes-de-Souza, Gabrielle Lacerda de Souza Gomes-Reis, Marina Ferreira-Costa, Carolina Medina Coeli da Cunha, Matheus Augusto Patrício de Almeida, Vanessa Estato, Kauê Francisco Corrêa Souza e Souza, Francisco da Silva dos Santos, Maria Alice dos Santos Mascarenhas Brito, Patrícia Novaes Soares, Wilza Arantes Ferreira Peres, Roland Immler, Matteo Napoli, Patrícia Torres Bozza, Hugo Caire de Castro-Faria-Neto, Markus Sperandio, Adriana Ribeiro Silva, Cassiano Felippe Gonçalves-de-Albuquerque

**Affiliations:** 1 Immunopharmacology Laboratory, Federal University of State of Rio de Janeiro, Rio de Janeiro, Brazil; 2 Post-Graduation Program in Molecular and Celular Biology, Federal University of State of Rio de Janeiro, Rio de Janeiro, Brazil; 3 Immunopharmacology Laboratory, Oswaldo Cruz Institute, Fiocruz, Rio de Janeiro, Brazil; 4 Post-Graduation Program in Molecular and Celular Biology, Oswaldo Cruz Institute, Fiocruz, Rio de Janeiro, Brazil; 5 Post-Graduation Program in Neuroscience, Fluminense Federal University, Rio de Janeiro, Brazil; 6 Department of Nutrition and Dietetics - Josué de Castro, Nutrition Institute, Federal University of Rio de Janeiro, Rio de Janeiro, Brazil; 7 Institute of Cardiovascular Physiology and Pathophysiology, Biomedical Center, Ludwig-Maximilians-University Munich, Munich, Germany

**Keywords:** docosahexaenoic acid, omega 3, microcirculation, neuroinflammation, intravital microscopy, neutrophils, clp

## Abstract

**Background:**

Sepsis is a life-threatening condition characterized by organ dysfunction resulting from a dysregulated host response to infection. Sepsis induces systemic inflammation and increases adhesion molecule expression and activation, promoting leukocyte adhesion to the endothelium. In addition, sepsis leads to the disruption of vascular integrity with fluid leakage and migration of leukocytes across the compromised endothelial barrier, leading to organ damage. Bioactive food compounds such as DHA, an essential omega 3 polyunsaturated fatty acid (PUFA) in the Mediterranean Diet (MedDiet), are known for their anti-inflammatory and pro-resolving properties. Thus, the supplementation of DHA may affect sepsis development, protecting the host.

**Methods:**

To investigate the role of DHA in neutrophil function, we conducted flow chamber assays using isolated neutrophils from mice and humans treated with DHA. To assess whether similar effects occur *in vivo*, we performed intravital microscopy of the TNF-stimulated cremaster muscle. Finally, we employed the cecal ligation and puncture (CLP) model to evaluate the therapeutic potential of DHA in experimental sepsis, and we applied intravital microscopy to assess cerebral vascular perfusion and the cerebral microcirculation in septic mice.

**Results:**

We found a significant reduction in neutrophil rolling and adhesion in DHA-treated neutrophils compared to controls in flow chamber assays, which can be mechanistically explained by a substantial reduction in adhesion markers, such as PSGL-1, CD11a, and CXCR4. Next, we employed intravital microscopy in the mouse cremaster muscle, stimulating it with tumor necrosis factor, and found a significant reduction in leukocyte rolling and adhesion in DHA-treated mice, confirming the *in vitro* flow chamber results. We also used a CLP model of sepsis. We found that DHA treatment ameliorated CLP-related sepsis parameters, including mortality, clinical score, total leukocyte and neutrophil transmigration, cytokine levels in peritoneal exudate, plasma, and brain tissue, and lactate levels. DHA treatment also improved cerebral microcirculatory perfusion and exhibited anti-inflammatory and pro-resolving effects, reflected by increased plasma and brain tissue resolving D1 and D2 levels.

**Conclusion:**

Together, we identify DHA as a promising anti-inflammatory therapeutic agent that mitigates sepsis-related vascular dysfunction and prevents organ failure.

## 1 Introduction

Inflammation is fundamental for the organism’s defense system against infections and injuries. It triggers mechanisms that eliminate pathogens and facilitate tissue repair, helping restore the physiological balance and normal function at sites of infection or damage (Calder, 2013). However, when this response becomes dysregulated and excessive in the presence of a pathogen, it may proceed to sepsis. Sepsis is a life-threatening condition characterized by a systemic inflammatory response to infection, leading to organ dysfunction (Arora et al., 2023). The transition from a beneficial inflammatory response to sepsis involves a failure to regulate the inflammatory process, resulting in an overwhelming release of pro-inflammatory cytokines, known as a “cytokine storm,” which exacerbates tissue damage and can result in multi-organ failure (Carcillo and Shakoory, 2024).

An intense inflammatory response is associated with significant alterations in the microcirculation, driven by mechanisms such as endothelial dysfunction, glycocalyx degradation, altered blood cell rheology (reduced red blood cell deformability), an imbalance between vasodilatory and vasoconstrictive mediators, and impaired autoregulation (Guven et al., 2020; Maneta et al., 2023). In sepsis, these changes often lead to disrupted microcirculatory perfusion, marked by decreased capillary density, restricted red blood cell (RBC) flow, and the formation of microthrombi (Guven et al., 2020; Maneta et al., 2023). Identified as a crucial endogenous process, the resolution of inflammation protects host tissues from the potential of prolonged or excessive inflammation that may become chronic (Panigrahy et al., 2021).

Bioactive compounds in plants and foods are essential for promoting health and preventing disease. Docosahexaenoic acid (DHA), an omega-3 fatty acid, has been widely recognized for its health benefits, particularly in the context of the Mediterranean diet (MeDiet) (Jerab et al., 2025). The Mediterranean diet emphasizes fruits, vegetables, whole grains, legumes, nuts, and healthy fats like olive oil and fish. It is a rich source of bioactive compounds, including polyphenols, flavonoids, and omega-3 fatty acids, such as DHA (Syren et al., 2022). The resolution of inflammation can be triggered by n-3 polyunsaturated fatty acids (PUFAs), which partially exert their effect through their conversion into bioactive lipid mediators called specialized pro-resolving mediators (SPMs) (Serhan, 2014). DHA is the primary precursor for the biosynthesis of the anti-inflammatory SPMs resolvins, protectins, and maresins (Ferreira et al., 2022). Besides SPMs, n-3 PUFAs have demonstrated significant anti-inflammatory properties (Calder, 2016; Calder et al., 2017), as they compete with arachidonic acid (ARA) as a substrate for cyclooxygenase (COX) and lipoxygenase (LOX) enzymes, decreasing the production of inflammatory eicosanoids like prostaglandin E2 (PGE2) and leukotriene B4 (LTB4), a process termed “lipid-mediator class switching” (Serhan et al., 2014). Furthermore, n-3 PUFAs modulate the expression of genes involved in inflammation by influencing transcription factors like NF-κB and PPAR-γ (Huang et al., 2022; Yang et al., 2024). n-3 PUFAs have been shown to reduce the expression of adhesion molecules (Shibabaw, 2021), including vascular adhesion molecule 1(VCAM-1) and intercellular adhesion molecule 1 (ICAM-1) (Abdolahi et al., 2021; Eslami Abriz et al., 2024), both essential for the recruitment of inflammatory cells to sites of inflammation. In addition to these mechanisms, DHA has also been shown to enhance the immunosuppressive function of neonatal myeloid-derived suppressor cells (MDSCs) via PPARγ-mediated fatty acid uptake and mitochondrial ATP production (Cui et al., 2024). Notably, passive transfer of MDSCs or perinatal DHA supplementation was able to counteract the pro-inflammatory effects of maternal circadian disruption on neonatal immune responses (Cui et al., 2024). Neutrophil recruitment involves a sequential molecular adhesion and activation cascade initiated by endothelial selectins (E−/P-selectins) binding to neutrophil ligands like PSGL-1, enabling initial tethering and rolling under shear forces. Chemoattractants and DAMPs activate neutrophil GPCRs, leading to integrin activation and firm adhesion via ICAM-1 (Pruenster et al., 2023). Guided by chemokine gradients, neutrophils crawl along the endothelium before transmigrating through endothelial junctions or non-junctional sites (Masgrau-Alsina et al., 2020). We have previously demonstrated the ability of omega-9 fatty acids to mitigate inflammation in a cecal ligation and puncture (CLP)-induced sepsis model with reduced cytokine levels, bacterial load, and leukocyte trafficking (Goncalves-de-Albuquerque et al., 2016; Medeiros-de-Moraes et al., 2018).

In this study, we aim to evaluate the effects of a single dose of DHA pretreatment, both *in vitro* and *in vivo,* on inflammation and experimental sepsis in mice. Specifically, we focus on its impact on mouse survival, leukocyte trafficking, microcirculatory function, and inflammatory mediator production.

## 2 Methods

### 2.1 Neutrophil isolation

Neutrophils were isolated from the bone marrow of C57BL/6 mice using a previously published protocol (Swamydas et al., 2015). For the isolation of neutrophils in humans (performed according to the approval of the Ludwig Maximilians-University of Munich - LMU Ethical Committee #611-15), we used the Polymorphprep protocol. In brief, after collecting the blood, 3 mL was transferred to a 15 mL Falcon tube with an additional 3 mL of Dulbecco’s PBS and homogenized with a pipette. Then, 6 mL of the diluted blood was carefully layered and centrifuged at 400 *g* for 30 min at 20 °C in a centrifuge without brake. The mononuclear cell layer was removed and placed in a new Falcon tube, and 3 times the volume of Hank’s solution was added for washing from the isolated cells, which were then centrifuged again at 400 g for 10 min. After counting, both murine and human cells were incubated with DHA (DHA sodium salt, D8768, Sigma-Aldrich®) 1 × 10^6^ cells in100 μM DHA in normal saline for 3 h at 37 °C and 5% CO2.

### 2.2 Flow cytometry

For analysis of cell viability, 1 × 10^6^ isolated neutrophils were incubated with DHA 100 uM for 3 h, and then the Zombie Yellow Fixable Viability Kit (BioLegend) was used according to manufacturer instructions. For the expression of adhesion molecules, neutrophils were identified in bone marrow as CD45^+^CD11b^+^Ly6G^high^ or CD45^+^CD11b^+^Gr-1^high^ after treatment. The cells were stained with antibodies against CXCR4, PSGL-1, integrin α4, CD11a, CD11b, and CD44 for 20 min at room temperature. All primary antibodies were used at a final 5 μg/mL concentration. For further details, see supplemental Methods. All experiments were conducted using a Beckman Coulter Gallios flow cytometer. Data were analyzed using FlowJo software (Tree Star, Ashland, OR).

### 2.3 Flow chamber assay

Ibidi flow chambers (µ-Slides VI 0.1) were coated with a combination of rmE-selectin (CD62E Fc chimera; 20 mg/mL; R&D Systems®), rmICAM-1 (ICAM-1 Fc chimera; 15 mg/mL; R&D Systems®) and rmCXCL1 (15 mg/mL; Peprotech®) or rhE-selectin (20 mg/mL; R&D Systems®), rhICAM-1 (15 mg/mL; R&D Systems®) and rhCXCL8 (15 mg/mL; Preprotech®), respectively for 3 h at room temperature and then blocked with 5% casein (Sigma-Aldrich®) overnight at 4 °C. For superfusion assays, isolated neutrophils were resuspended in HBBS and treated with DHA or saline were perfused through the flow chamber with a high-precision syringe pump (Harvard Apparatus) at a shear stress level of 2 dyne/cm^2^ and recorded for 10 min. For the crawling assay, cells were placed in the chamber for 3 min before perfusion was started with HBSS medium and video was recorded for 20 min. Experiments were conducted on a ZEISS AXIOVERT 200 microscope with a ZEISS LD Plan-neofluor objective (×20, 0.4 NA: and a SPOT RT ST Camera). MetaMorph software generated films for subsequent analysis using FIJI software as already described (Zengel et al., 2011; Schindelin et al., 2012).

### 2.4 Animals

Swiss Webster male mice (*Mus musculus*), aged between 3 and 4 weeks, obtained from the ICTB, were used in the experiments. All procedures described in this project adhere to ethical principles and current national legislation and are approved by the CEUA/IOC License L-027/2022-A2.

### 2.5 Cecal ligation and puncture (CLP)

The CLP sepsis model was performed as described previously (Goncalves-de-Albuquerque et al., 2018). Three hours before the surgical procedure, a single dose of 100 μL of ultrapure DHA (CAS No.: 81926-93-4; Sigma-Aldrich®) (200 mg/kg) or Saline was administered i.p. Mice were anesthetized with ketamine (100 mg/kg) and xylazine (10 mg/kg) administered i.p. prior to CLP surgery. The surgical procedure was performed on a heated pad, and mice were kept on it until they fully recovered from anesthesia. Immediately after the procedure, 1 mL of sterile saline was administered subcutaneously to replace fluid and heat lost. Animals remained on the heating pad until complete recovery and were then returned to their cages with free access to food and water. The clinical score for sepsis severity was assessed 24 hours post-CLP, mice using a scoring system where higher scores indicate greater severity, as reported before (Goncalves-de-Albuquerque et al., 2018). The evaluation included piloerection, curved posture, gait abnormalities, seizures, lethargy, respiratory rate, lacrimation, grip strength, fecal changes, body tone, and temperature fluctuations. Each animal received a score ranging from 1 to 11, with classifications as follows: 1-3 (mild sepsis), 4-7 (moderate sepsis), and 8-11 (severe sepsis). Humane endpoints were monitored at 1, 6, and 20 h after the procedure using a clinical scoring system. Mice with scores above 10 were immediately anesthetized to prevent suffering. Under our experimental conditions, most mice were categorized as having moderate sepsis. Treatment administration and surgery were performed by investigators blinded to group assignment.

### 2.6 Peritoneal lavage

Peritoneal lavage was performed as described previously (Medeiros-de-Moraes et al., 2018). Mice were euthanized 24 h post-surgery using isoflurane (Cristália). Under sterile conditions, the peritoneal cavity was flushed with 3 mL of cold sterile saline inside a laminar flow hood. The peritoneal lavage was used to assess bacterial growth by counting CFUs, cytokine analysis and differential cell count. Red blood cells were lysed with Turk’s solution (2% acetic acid), and total cell count was determined using a Neubauer hemocytometer. Differential leukocyte counts were performed on cytocentrifuged stained with panoptic (Laborclin).

### 2.7 CFU

Bacterial quantification in the peritoneal lavage was carried out 24 h post CLP. The lavage fluid was diluted 1:10,000 in sterile PBS, and 10 µL of the diluted samples were plated on TSA medium (Difco Laboratories®) in sterile Petri dishes. All steps were performed under aseptic conditions. The plates were incubated at 37 °C for 18 h, after which colony-forming units (CFUs) were counted.

### 2.8 Cytokine assessment by ELISA

The concentrations of tumor necrosis factor (TNF), IL-1β, IL-6, IL-10, CXCL1, MCP1, and BDNF in the supernatants of peritoneal fluid, plasma, or brain lysates were determined using enzyme-linked immunosorbent assay (ELISA) kits (DuoSet, R&D Systems, Minneapolis, MN, United States) following the manufacturer’s protocols. Levels of resolvins D1 and D2 were quantified using enzyme immunoassay (Cayman Chemical, MI, United States) according to the manufacturer’s instructions.

### 2.9 Lactate quantification

Lactate levels were measured using the Accutrend Plus Monitor (COBAS, Roche®). For the analysis, 30 μL of blood was applied to the device equipped with lactate-specific test strips. The results were expressed in millimoles per liter (mM/L).

### 2.10 Cremaster intravital microscopy experiments

Three hours prior to the procedure, mice were administered an intraperitoneal (i.p.) injection of 100 μL of DHA (200 mg/kg; CAS No.: 81926-93-4; Sigma-Aldrich®) or an equivalent volume of saline. Two hours before intravital microscopy, an intra-scrotal injection of 500 ng of TNF-α (R&D Systems) was administered. Mice were anesthetized and prepared for intravital microscopy following previously established protocols (Napoli et al., 2024). Intravital microscopy was performed using a conventional brightfield microscope (Zeiss Axio Examiner.D1) equipped with a ×40 objective (0.75 NA, water immersion), an Axiocam 702 CMOS camera and a Colibri epifluorescence LED excitation light source (Zeiss®, Germany). Blood flow velocity was measured with fluorescent beads (Flouresbrite® YG Microspheres, 1 µm). Post-capillary venules observed ranged from 20 to 50 μm in diameter. Blood samples were collected, and white blood cell counts were determined using the ProCyte Dx Hematology Analyzer (IDEXX, Westbrook, USE-ME). All analyses were conducted using Fiji software (Schindelin et al., 2012).

### 2.11 Brain intravital microscopy experiments

Animals were anesthetized and placed in a stereotaxic apparatus. Using a high-speed drill, a cranial window was created over the right parietal bone, with the dura mater and arachnoid layers removed to expose the cerebral microvasculature. The caudal vein was cannulated to administer dyes and additional anesthetic when necessary. Animals were positioned under a fixed-stage vertical intravital microscope equipped with an LED lamp, connected to a Zeiss Axiocam, with image processing conducted using ZEN lite software (Zeiss®). For the experiments we used a water immersion ×20 objective, yielding a total magnification of ×200. The microvascular surface of the brain was visualized through intravenous injection of fluorescein isothiocyanate (FITC-Dextran, CAS no 60842468, Sigma-Aldrich®, St. Louis, MO) and epi-illumination at 460–490 nm. Leukocytes were labeled with Rhodamine 6G (CAS no 989388, Sigma-Aldrich®, St. Louis, MO) and visualized via epi-illumination at 536–556 nm excitation. Leukocyte-endothelium interactions were assessed by analyzing at least five post-capillary venules. Rolling leukocytes were quantified as the number of cells moving slower than red blood cells through the venular segment in 1 min. Adherent leukocytes were defined as immobile and attached to the endothelial wall for 1 min and were quantified based on the total leukocyte count and vessel area (mm^2^). All analyses were performed using ZEN lite software (Zeiss®).

### 2.12 Laser speckle imaging

Cerebral blood flow was measured using Laser Speckle Contrast Imaging (LSCI) (PeriCam PSI System, Perimed®, Sweden). To assess cerebral blood flow 24 h after CLP surgery, animals were anesthetized, the scalp was incised, and the superficial skin was removed, exposing the skull. A region of interest (ROI) approximately 2 cm^2^ in size was marked on the skull to evaluate cerebral blood flow. Blood flow was analyzed by capturing 19 images per second, and relative cerebral blood flow was quantified using Perisoft software (PeriCam PSI System, Perimed®, Sweden), expressed in arbitrary perfusion units (APU).

### 2.13 Statistical analyses

Data were analyzed using GraphPad Prism 8.0.1 software. Results are expressed as mean ± standard deviation (SD) or standard error of the mean (SEM). Statistical differences were determined using One-Way ANOVA followed by Bonferroni’s *post hoc* test for multiple comparisons. Kaplan-Meier survival curves were analyzed using the Log-rank (Mantel-Cox) test. A p-value ≤0.05 was considered statistically significant.

## 3 Results

### 3.1 DHA treatment reduced the expression of adhesion molecules and leukocyte rolling and adhesion *in vitro*

**FIGURE 1:**
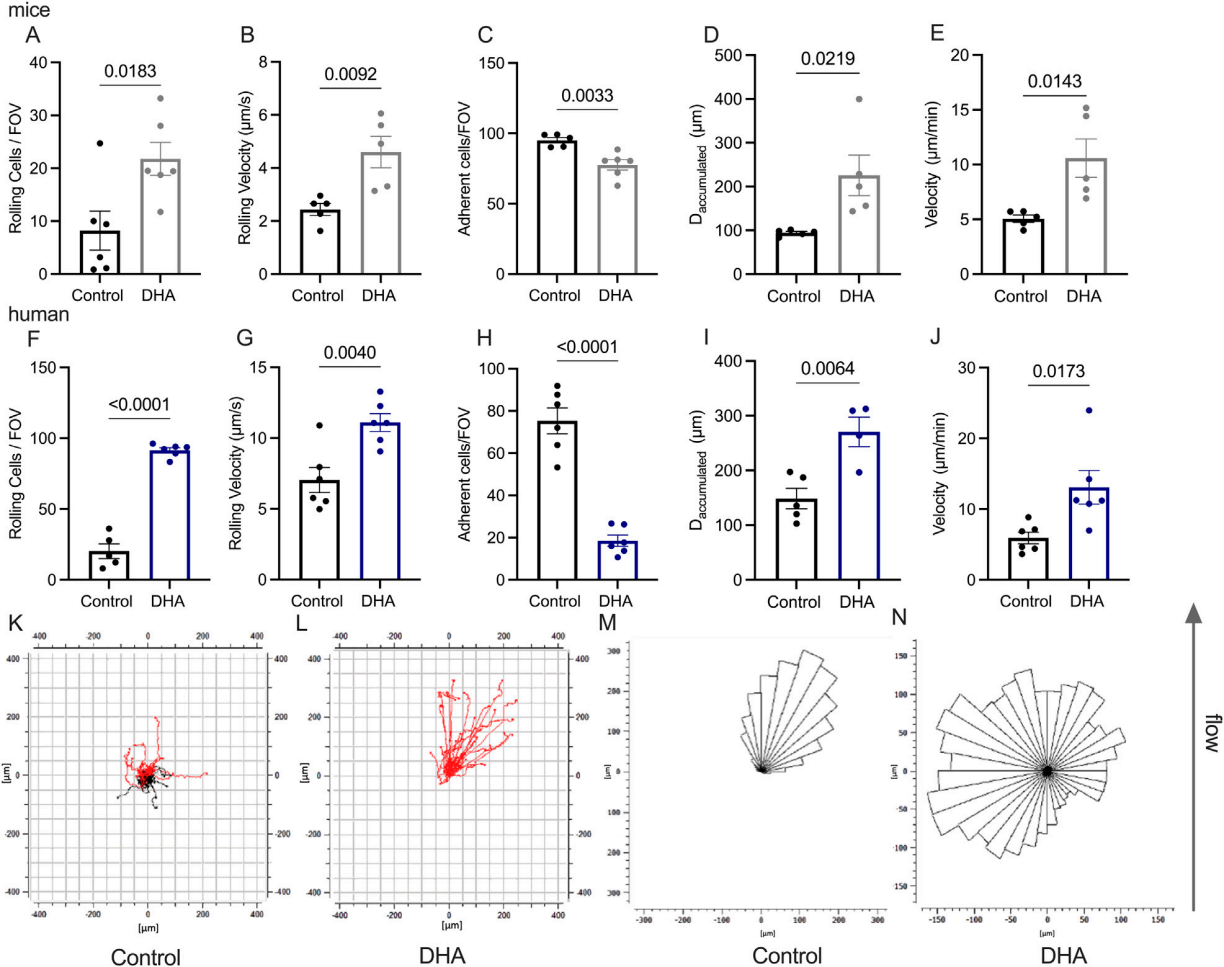
DHA treatment inhibited neutrophil recruitment *in vitro* in a flow chamber assay. 1 × 10^6^ neutrophils isolated from bone marrow were treated for 2 h with 100 µM DHA. In panels **(A–C)**, the number of rolling cells, rolling velocity, and adhered cells were observed over 10 min under flow conditions. The crawling phenomenon was analyzed, and the distance traveled was analyzed in panel **(D)** and the crawling velocity in panel **(E)**. The same experiment was conducted with isolated human neutrophils to determine if human neutrophils would maintain the observed effects, as shown in figures **(F-J)**. **(K-L)** Representative graph of single human neutrophils migration. The black lines indicate adhered cells and those crawling against the flow. **(M,N)** Rose plot diagrams represent migratory crawling trajectories of human neutrophils. The values represent means with SEM, and SEM is presented with vertical bars; *p < 0.05, **p < 0.01, ***p < 0.005, ****p < 0.001, as determined by two-tailed unpaired Student’s t-tests. n = 4-7.

The interaction between activated endothelial cells and circulating neutrophils initiates a precisely regulated sequence of events known as the leukocyte recruitment cascade, consisting of rolling, adhesion, postarrest modifications, and transendothelial migration (Nemeth et al., 2020). After observing impaired leukocyte recruitment in the cremaster venules, we investigated the potential role of DHA in this process. To study the potential role of DHA in this process, we conducted flow chamber experiments to assess neutrophil rolling, adhesion, and crawling. Flow chambers were coated with a combination of recombinant mouse ICAM-1, E-selectin, and CXCL1. We observed that murine bone marrow neutrophils incubated with DHA for 3 h displayed an increased number of rolling cells compared to control neutrophils. In addition, rolling velocities were increased in neutrophils treated with DHA (Figures 1A, B). Next, we investigated neutrophil adhesion under flow and detected fewer adherent neutrophils upon DHA treatment (Figure 1C).

Given the significant impact of DHA on leukocyte rolling and adhesion, we next evaluated post-arrest modifications under flow including cell spreading and crawling. The flow chambers were coated with murine E-selectin, ICAM-1, and CXCL1. the neutrophils were perfused into flow chambers and allowed to settle down and firmly adhere before flow was applied through a high-precision pump, and neutrophil crawling behavior was assessed under shear flow. DHA-treated neutrophils exhibited greater cumulative migration distance and higher crawling velocity than respective control neutrophils (Figures 1D, E). Having found a significant influence of DHA on murine neutrophil recruitment, we next examined the recruitment behavior of human neutrophils. Human neutrophils were isolated from peripheral blood and incubated with DHA, and also exhibited an increased number of rolling cells and higher rolling velocity compared to control neutrophils in flow chambers coated with a combination of recombinant human E-selectin, ICAM-1 and CXCL8, indicating altered rolling dynamics following DHA exposure (Figures 1F, G). Under flow conditions, we observed a marked reduction in the number of adherent human neutrophils following DHA treatment (Figure 1H), which exceeded the reduction found in mouse neutrophils (Figure 1C), suggesting that DHA has a strong effect on adhesion in human neutrophils. As in murine neutrophils, DHA treatment also altered the crawling behavior of human neutrophils. DHA-treated cells exhibited increased crawling distance and velocity under shear flow (Figures 1I, J). Notably, DHA-treated neutrophils migrated toward the flow direction implying reduced resistance to shear stress. In contrast, control neutrophils displayed a crawling pattern which seemed to be influenced less by shear forces (Figures 1K–N), as reported earlier (Immler et al., 2022). These experiments demonstrated that DHAincubated neutrophils exhibited reduced adhesion and increased rolling velocity under flow conditions, along with a more unstable crawling phase compared to control cells. N-3 fatty acids inhibit the expression of adhesion molecules (Calder, 2017). To assess whether DHA treatment directly influences neutrophil adhesion molecule expression, we isolated neutrophils from bone marrow and treated them with 100 μM DHA for 3 h. Then, we investigated its potential to modulate expression of adhesion-relevant molecules such as PSGL-1, CD11a, CD11b, CD44, alpha4 integrin, and CXCR4, by flow cytometry. Following DHA treatment, isolated neutrophils exhibited reduced expression of CXCR4, PSGL-1, and CD11a compared to control-treated neutrophils, while CD11b CD44 and alpha4 integrin expression were not different between the groups (Supplementary Figure S1).

### 3.2 Systemic DHA treatment reduced neutrophil recruitment *in vivo*

**FIGURE 2:**
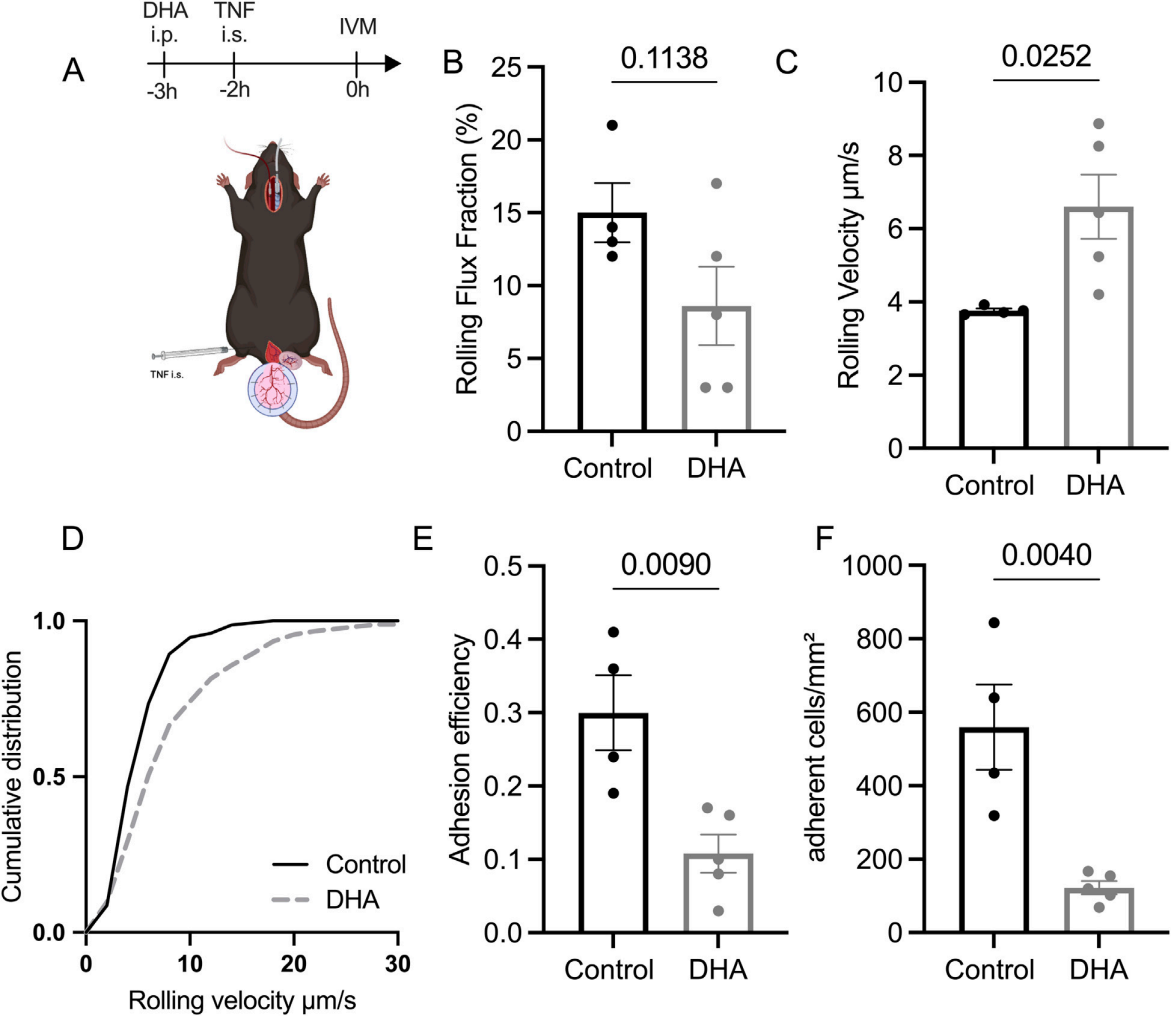
DHA reduced microcirculation inflammation in a murine microvascular inflammation model. **(A)** Mice received DHA 200 mg/kg i.p. 3 h prior and 500 ng of TNF via intrascrotal injection 2 before intravital microscopy of postcapillary venules of the mouse cremaster muscle, **(B)** Leukocyte rolling flux fraction, **(C)** Rolling Velocity, **(D)** Cumulative velocity distribution was calculated from at least 30 rolling cells per group, **(E)** Number of adherent cells per mm^2^, **(F)** Adhesion efficiency was calculated from at least 8 venules in 4 or 5 mice. The values represent means with SEM; *p < 0.05, **p < 0.01; ***p < 0.005, ****p < 0.001, as determined by two-tailed unpaired Student’s t-tests. n = 4 (control)-5 (DHA).

To assess whether DHA treatment alters leukocyte recruitment *in vivo*, we employed intravital microscopy in an acute neutrophildriven inflammation model of the mouse cremaster muscle. To do so, we first applied a single dose of DHA i.p. at 200 mg/kg 1 h later, we injected 500 ng of TNF intrascrotally and surgically exteriorized the cremaster muscle 2 h later for intravital imaging (Figure 2A). In the inflamed cremaster muscle microcirculation, we observed a decreased leukocyte rolling flux fraction and increased leukocyte rolling velocity in DHA-treated mice compared with untreated mice (Figures 2B–D). Furthermore, we found a reduction in both leukocyte adhesion (cells/mm^2^) (Figure 2E) and leukocyte adhesion efficiency (the ratio of adherent cells/mm^2^ to systemic leukocyte count) (Figure 2F) in mice treated with DHA. Of note, microvascular and hemodynamic parameters in the intravital microscopy experiments did not differ between groups (Supplementary Table S2). These findings demonstrate a strong anti-inflammatory effect of DHA on leukocyte recruitment during the acute inflammatory response.

### 3.3 DHA treatment inhibited mortality and clinical score in CLP sepsis model

**FIGURE 3:**
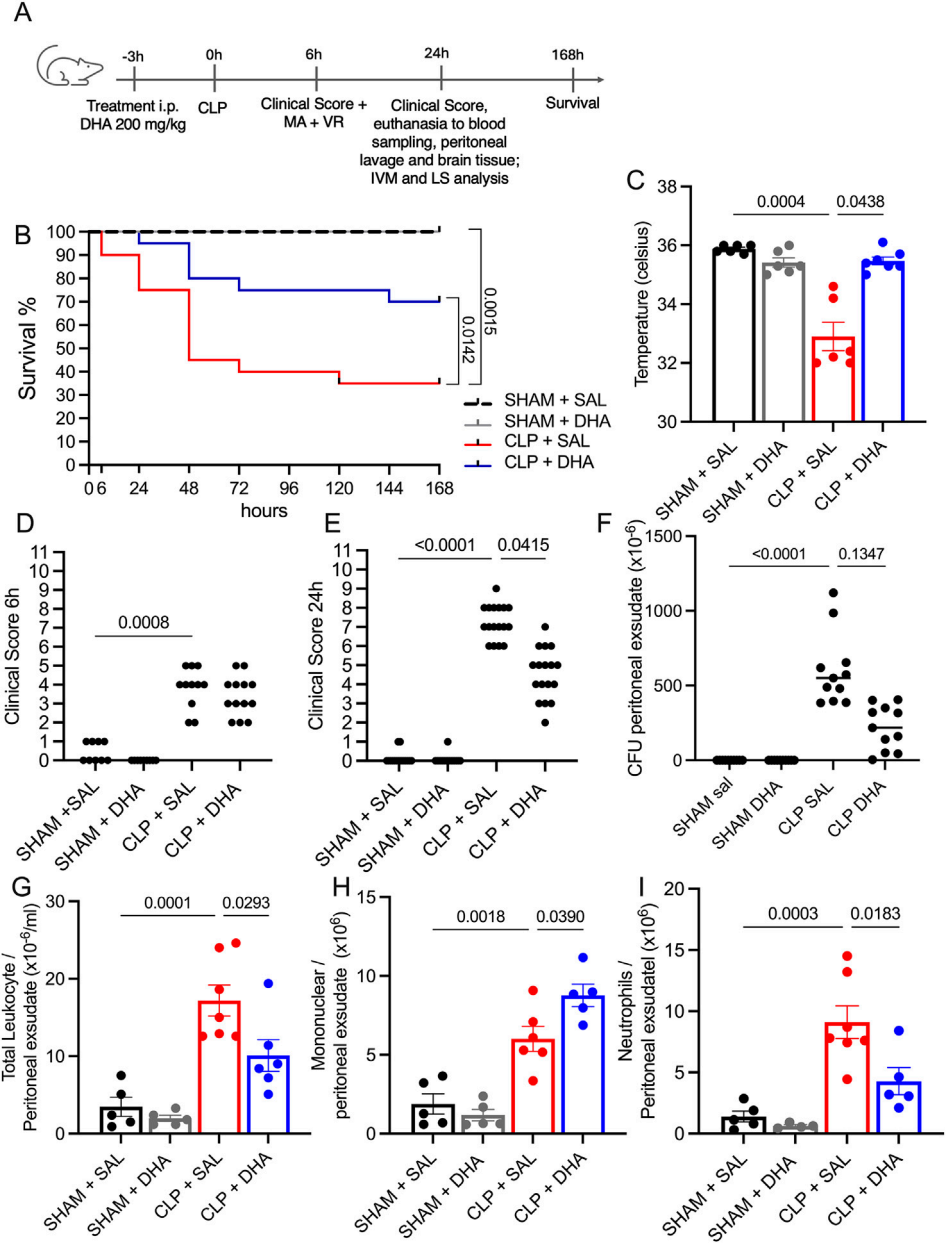
DHA treatment reduced sepsis-related markers in septic mice. **(A)** Timeline of analyses performed in the experimental design. Abbreviations: CLP, cecal ligation and puncture; Sham, sham-operated control; i.p., intraperitoneal MA–Meropenem antibiotic (10 mg/kg), VR–volemic reposition (saline + glucose 20%), IVM–intravital microscopy, LS–laser speckle. **(B)** Survival curve of animals pre-treated with DHA 200 mg/kg. The animals were observed twice daily for the first 48 h and then until the seventh day. The Kaplan-Meier survival curve was generated with the Log-rank (Mantel-Cox) test, n = 20. **(C)** Temperature of the animals 24 h after CLP treated with DHA 200 mg/kg n = 5-7. **(D)** Clinical score 6 h after surgery, **(E)** 24 h after surgery. n = 6-10 (Sham), 10-14 (CLP). **(F)** Bacterial growth in the peritoneal lavage of CLP animals treated with DHA 200 mg/kg. The lavage was diluted to 10^4^ and plated on TSA plates, then incubated for 24 h at 37 °C. n = 8-11. **(G)** Total leukocytes that migrated to the peritoneal lavage. **(H)** Number of monocytes and **(I)** neutrophils present in the peritoneal lavage. n = 5-7. Data are presented as mean ± SD and analyzed by one-way ANOVA with a Dunnetts’s multiple comparison test **(B–E)** and one-way ANOVA with Bonferroni multiple comparison test **(F–H)**. *p < 0,05, **p < 0,01; ***p < 0.005, ****p < 0.001. Data represent a representative experiment (from three independent experiments).

To evaluate the protective effects of DHA treatment in a sepsis model, we administered 200 mg/kg of DHA 3 h before CLP (Figure 3A). The primary outcome measured was the potential of DHA to prevent mortality. In the control group, the survival rate was 35% after 7 days, with the highest mortality occurring between days 1 and 2 post-CLP. In contrast, DHA treatment improved survival, with 70% of the mice surviving CLP-induced sepsis (Figure 3B). Body temperature is a key predictor of mortality in septic mice (Mai et al., 2018). In this study, the CLP saline group exhibited a significant drop in skin body temperature to 32.9 °C. In contrast, DHA-treated mice reversed this effect, maintaining a temperature of 35.4 °C (Figure 3C). Clinical scores were evaluated at 6 and 24 h post-CLP. Early signs of sepsis were observed at 6 h (Figure 3D), while the infection peaked at 24 h. Notably, for the 24-h time point, DHA treatment alleviated sepsis-related sickness behavior (Figure 3E). Next, bacterial CFU counts from peritoneal exudate were measured 24 h after CLP. A significant increase in bacterial CFU counts was observed in septic mice, while DHA treatment markedly reduced the bacterial load in the inflamed peritoneal cavity (Figure 3F). Septic mice exhibited higher leukocyte accumulation in the peritoneal cavity, particularly neutrophils, compared to the sham group (Figures 3G,H). Interestingly, DHA treatment suppressed peritoneal neutrophil recruitment but increased the number of monocytes and macrophages (Figure 3I). Given the critical role of macrophages in bacterial clearance and resolution of inflammation, these findings suggest a faster clearance of bacteria in the infected peritoneal cavity.

### 3.4 DHA treatment reduced cytokine levels in septic mice

**FIGURE 4:**
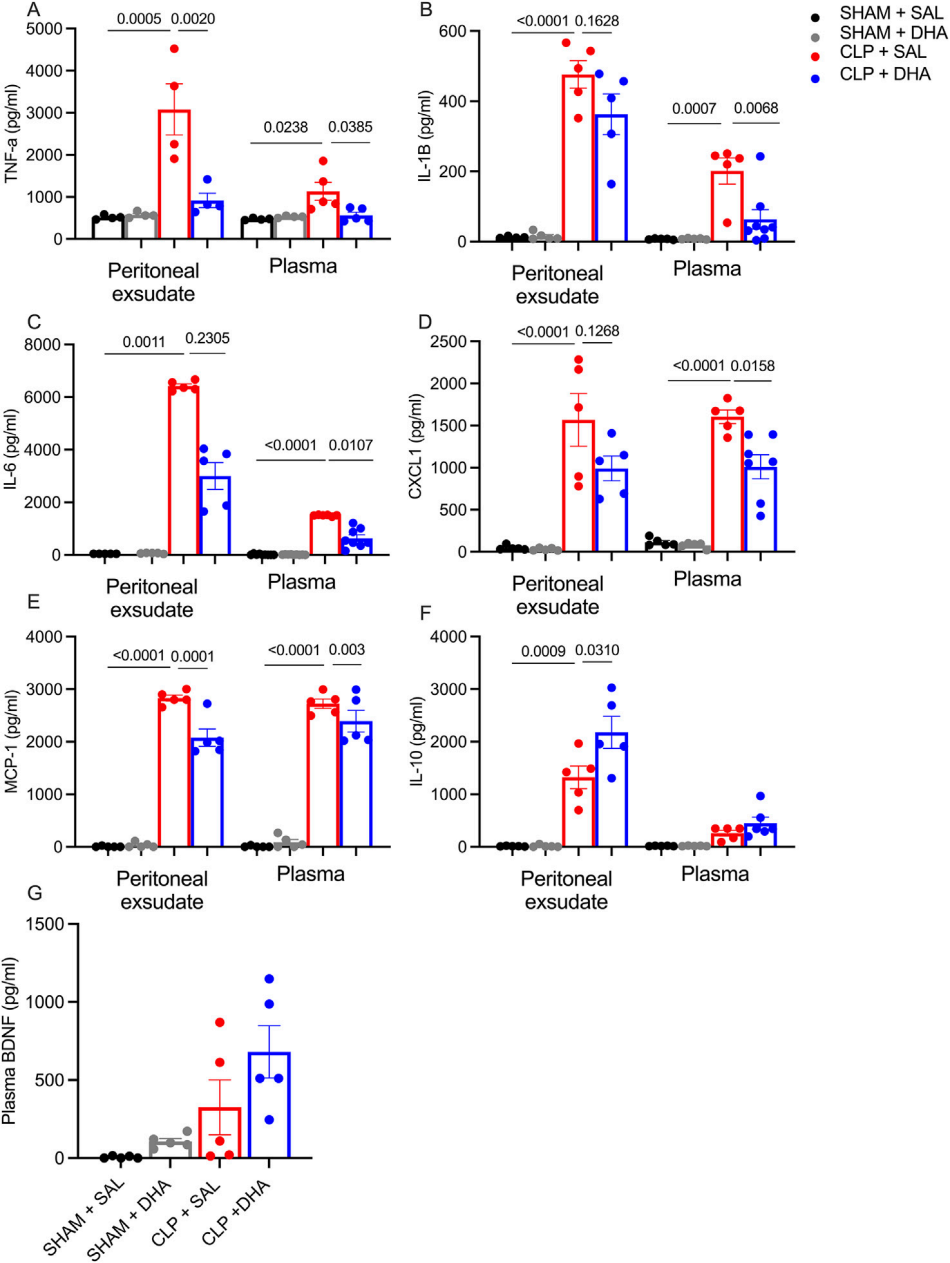
DHA decreased pro-inflammatory mediator production in septic mice. In animals treated with DHA at 200 mg/kg dose, cytokines were assessed 24 h after CLP in peritoneal exudate and plasma. Quantitative analysis of cytokines **(A)** TNF-ɑ, **(B)** IL-1β, **(C)** IL-6, **(D)** CXCL1, **(E)** MCP-1, **(F)** IL-10, and **(G)**. BDNF in peritoneal lavage and plasma measured by ELISA, expressed in pg/mL. The values represent means with SD, and SD is presented with vertical bars; *p < 0.05, **p < 0.01, ***p < 0.0005, ****p < 0.0001, as determined by one-way ANOVA with Bonferroni multiple comparison test. n = 4-7. Data represent a representative experiment (from three independent experiments).

**FIGURE 5:**
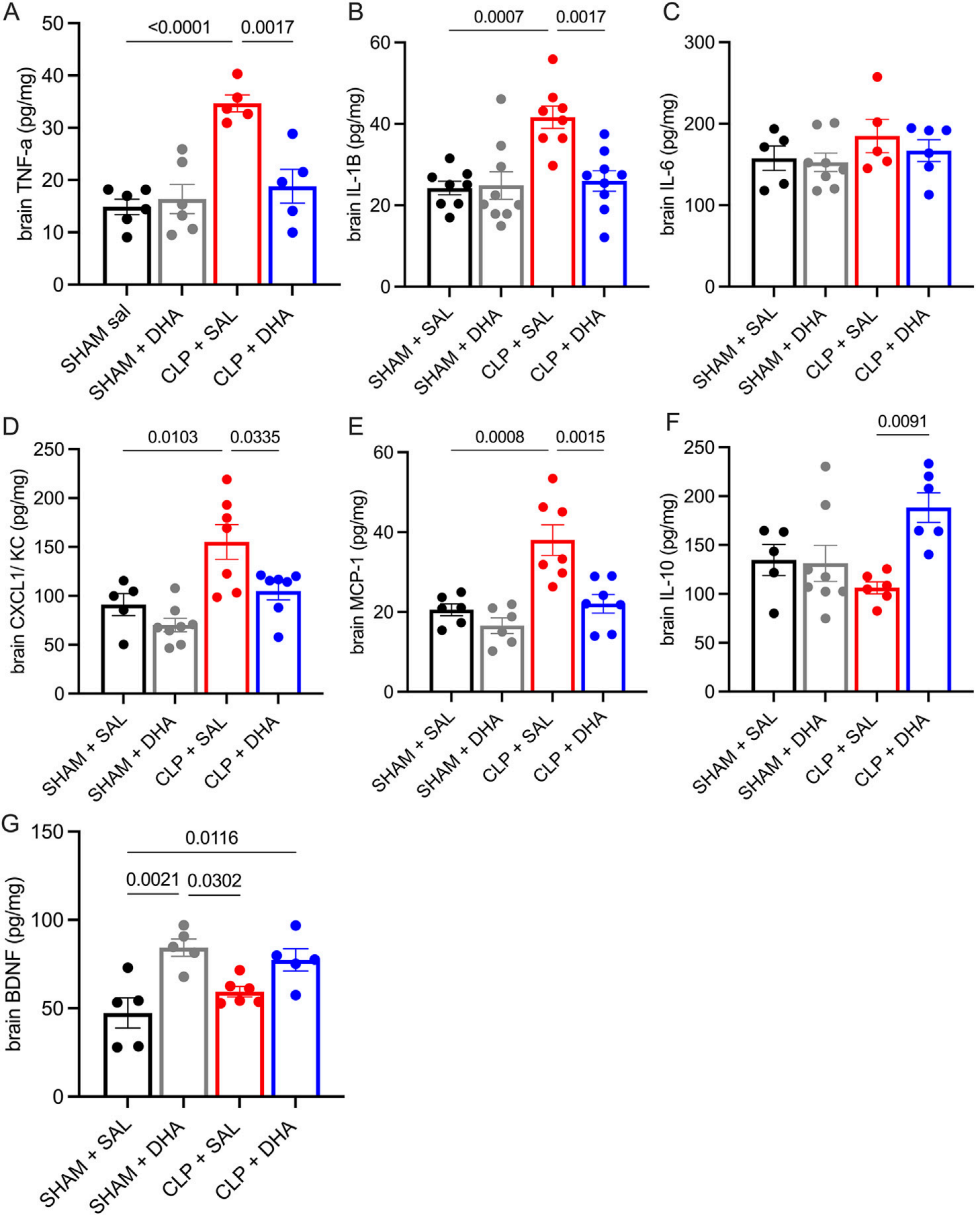
DHA ameliorates inflammation in the brains of septic mice. Evaluation of brain cytokines in animals treated with DHA 200 mg/kg 24 h after CLP. Quantitative analysis of cytokines **(A)** TNF-ɑ, **(B)** IL-1β, **(C)** IL-6, **(D)** CXCL1, **(E)** MCP1, **(F)** IL-10, and **(G)**. BDNF in peritoneal lavage measured by ELISA, expressed in pg/mg of brain tissue protein. The values represent means with SD; SD is presented with vertical bars; *p < 0.05, **p < 0.01, ***p < 0.005, ****p < 0.001, as determined by one-way ANOVA with Bonferroni multiple comparison test. n = 5-7. Data represent a representative experiment (from two independent experiments).

We aimed to determine whether DHA supplementation before CLP could reduce plasma and peritoneal cytokine levels. DHA influences transcription factors including AP-1 and NF-kB, which regulate expression of pro-inflammatory mediators (Zgorzynska et al., 2021). Compared to the Sham group, all analyzed cytokine levels were significantly elevated in the CLP group (Figure 4). In the peritoneal exudate, DHA treatment reduced TNF-α, IL-1β, IL-6, and MCP1 levels compared to the CLP saline group, while in plasma (Figures 4A–C,E), DHA lowered TNF-α, IL-1β, IL-6, and CXCL1 (Figures 4A–D) 24 h after CLP compared to CLP saline. DHA also showed significantly higher IL-10 (Figure 4F) levels in the peritoneal exudate than in the CLP saline group. Plasma brain-derived neurotrophic factor (BDNF), a key marker of DHA activity in the brain (Sugasini et al., 2020), was not different between the CLP DHA and CLP saline groups, suggesting that, despite DHA’s known neuroprotective properties, its effects in this context may not be mediated through systemic BDNF changes, highlighting the need to explore alternative mechanisms of action. Next, we assessed whether DHA treatment could attenuate brain inflammation by evaluating cerebral cytokine expression. Given that sepsis-associated encephalopathy induces widespread neuroinflammation, total brain lysates were analyzed to capture the global inflammatory response. However, this approach is limited by the loss of regional and cell-type specificity. In brain tissue, DHA treatment resulted in reduced levels of TNF-α, IL-1β, KC, and MCP-1 (Figures 5A,B,D,E), while IL-10 (Figure 5F) levels were elevated in the CLP-DHA-treated group. Cytokine IL-6 levels in the brain did not show significant alteration (Figure 5C). BDNF (Figure 5G) levels increased in the sham-treated group, but this effect was not maintained in the CLP group. These findings indicate that DHA supplementation modulates the inflammatory response by reducing pro-inflammatory cytokines and increasing IL-10, suggesting a protective role of DHA in CLP-induced systemic and brain inflammation.

### 3.5 DHA treatment increased resolvin levels

**FIGURE 6:**
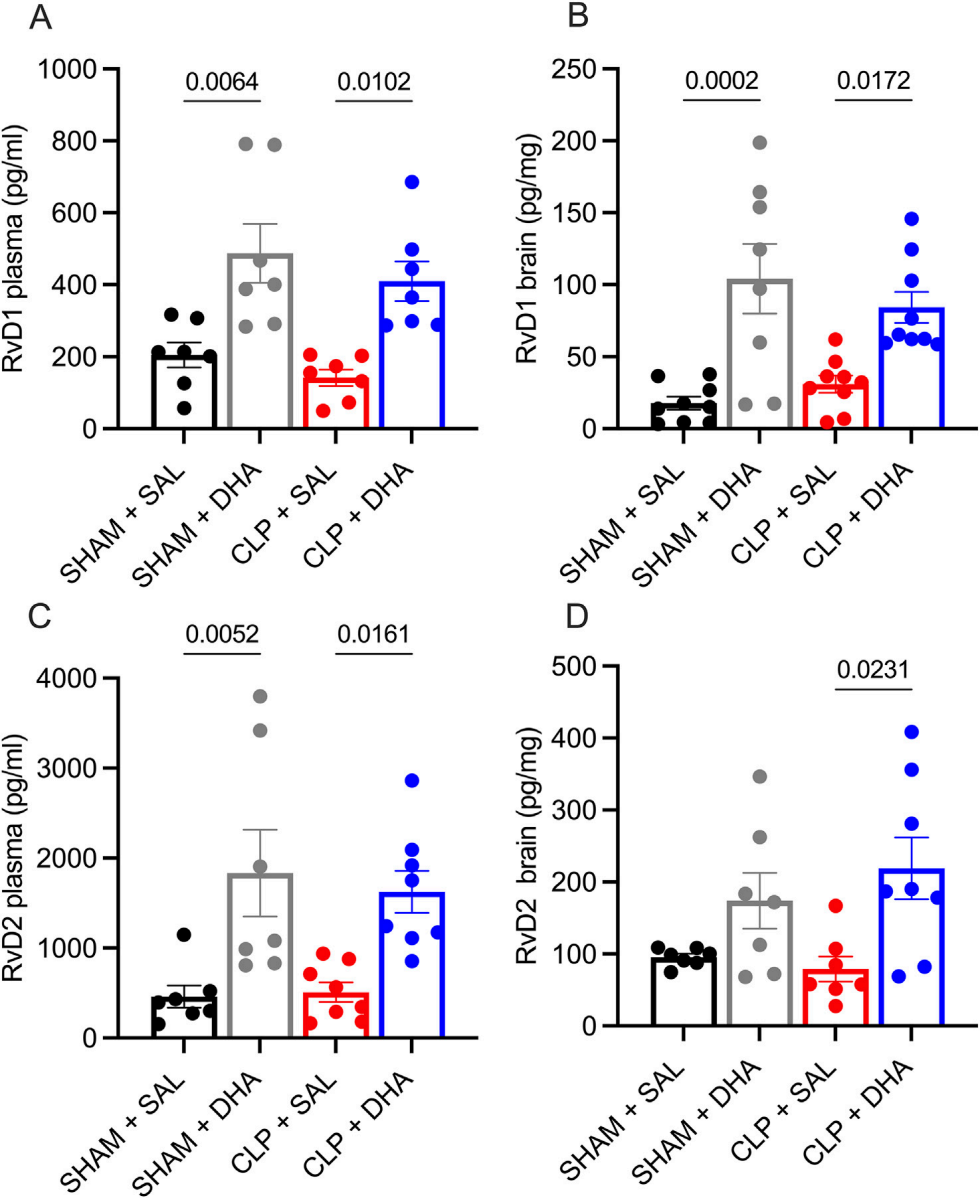
DHA induced the production of pro-resolutive mediators. Quantification of Resolvin D1 and D2 levels in the plasma and brain of CLP animals treated with DHA 200 mg/kg. **(A)** Plasma Resolvin D1, **(B)** Brain Resolvin D1, **(C)** plasma Resolvin D2, and **(D)** brain Resolvin D2. Values are expressed as means with SD, presented as vertical bars. Analysis was performed using one-way ANOVA with Bonferroni multiple comparison test. n = 5-10.

DHA-derived resolvins (RvD), key pro-resolving mediators, actively suppress inflammation by inhibiting leukocyte infiltration, enhancing macrophage phagocytosis, and regulating cytokine production. Through interactions with G-protein-coupled receptors, they promote tissue repair and resolution of inflammation, ensuring a controlled immune response and preventing chronic inflammation (Chiang and Serhan, 2020). To determine whether the reduction in inflammatory markers was associated with the production of specialized pro-resolving mediators, we quantified RvD1 and RvD2 levels. Despite administering a single DHA dose, a significant increase of RvD1 and RvD2 was observed in DHA-treated groups compared to controls in SHAM and CLP conditions in the plasma and brain tissue (Figure 6). These findings suggest that DHA treatment may facilitate resolution of inflammation by activating specific pro-resolving pathways, thereby contributing to the observed anti-inflammatory effects.

### 3.6 DHA improved cerebral perfusion and microcirculation in septic mice

**FIGURE 7:**
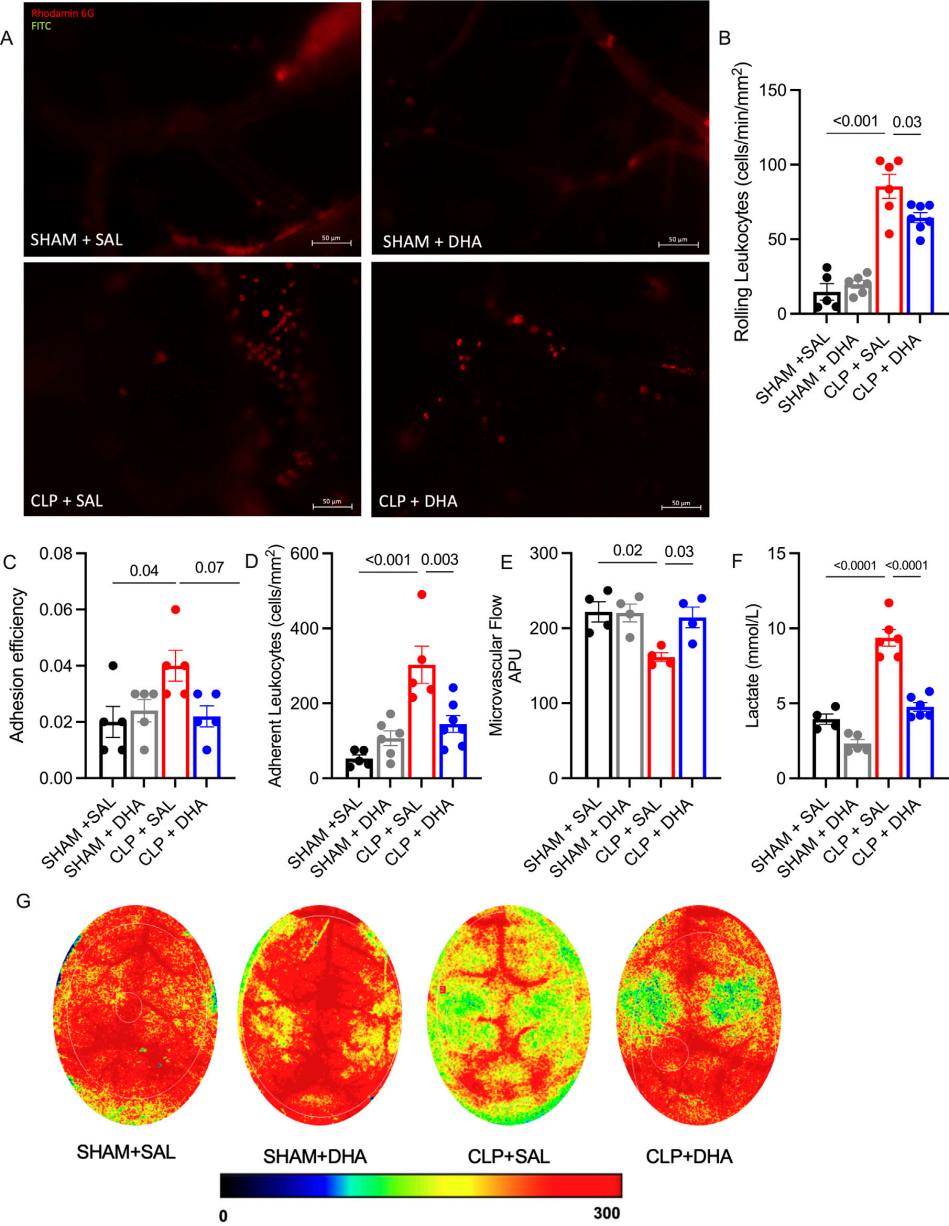
DHA improved microcirculation and perfusion in septic mice. Intravital microscopy analysis of cerebral microcirculation in animals induced with sepsis by CLP and treated with DHA 200 mg/kg. **(A)** Representative image of blood vessels under intravital microscopy for each group. **(B)** Rolling cells fraction per minute per mm^2^, **(C)** Adherent cells per mm^2^, **(D)** Leukocyte adhesion efficiency to the endothelium, **(E)** Lactate levels in plasma. **(F)** Blood flow intensity assessed by laser speckle imaging, expressed in Arbitrary Perfusion, and **(G)** Representative images of perfusion under laser speckle for each group 24 h after sepsis induction by CLP. *p < 0.05, ****p < 0.001, as determined by one-way ANOVA with Bonferroni multiple comparison test. N = 4-6. Data were calculated from at least 8 postcapillary venules from 4 to 5 mice.

Following our evaluation of DHA treatment on the inflammatory response in the inflamed mouse cremaster muscle microcirculation, we wanted to determine whether DHA also influences inflammatory processes, including microcirculatory parameters in the brain. The brain was selected for this, as DHA has been demonstrated to accumulate in the brain after application (Arellanes et al., 2020; Ali et al., 2024). First, we aimed to determine whether CLP-induced sepsis leads to the induction of leukocyte recruitment into the brain. To this end, we performed intravital brain imaging in mice 24 h after CLP. Compared to CLP saline, our analysis revealed a significant reduction in rolling leukocytes, measured per minute and per mm^2^ (Figure 7B), and a decrease in adherent leukocytes along the endothelium (Figures 7A,C) in the CLP DHA group. Additionally, the adhesion efficiency - calculated as the proportion of leukocytes adherent to the endothelium relative to the total circulating leukocytes - was partially reduced by DHA treatment compared to CLP saline (Figure 7D). These results suggest that DHA may exert anti-inflammatory effects by modulating leukocyte dynamics within the cerebral microcirculation, similar to its impact on the cremaster muscle tissue. Next, we used laser speckle imaging and assessed vascular perfusion in the brain following CLP. We found that septic mice exhibited a significant reduction in brain perfusion (Figures 7E,G), which was not observed in DHA-treated CLP mice. Impaired tissue perfusion is often associated with elevated lactate levels, a well-established clinical marker of sepsis. Consistent with the observed perfusion deficits, septic mice displayed increased lactate levels, while in DHA-treated animals lactate levels did not increase (Figure 7F).

## 4 Discussion

During sepsis, pathogens trigger the release of inflammatory mediators that activate leukocytes and endothelial cells. This activation leads to the upregulation of adhesion molecules on endothelial cells, which enhances their capacity to capture circulating leukocytes (Hotchkiss et al., 2016). In response to cytokine signaling from immune cells, the endothelium produces vasoactive compounds, inflammatory cytokines, and chemoattractants. Although these processes aid in controlling local infections, systemic endothelial activation can result in widespread complications, including microvascular thrombosis, increased capillary permeability, hypotension, tissue hypoxia, and tissue dysfunction (Dolmatova et al., 2021). Leukocyte transmigration across postcapillary venules occurs through adhesion molecules expressed on endothelial cells and their interactions with ligands on leukocytes (Gronloh et al., 2021). Key adhesion molecules include selectins, which mediate leukocyte capturing and rolling; integrins that facilitate firm adhesion, and ICAMs and VCAM-1, which act as integrin ligands to promote stable adhesion and facilitate leukocyte transmigration (Schmidt et al., 2013). This study investigated the anti-inflammatory properties of DHA, firtly focusing on neutrophil activation and migration and then leukocyte-endothelial interaction We demonstrated that DHA pre-treatment of isolated human and mouse neutrophils reduced adhesion, rolling, and crawling under flow *in vitro* and *in vivo*. Interestingly, DHA is associated with reduced migration in neutrophils treated with resolvin D1 by reducing actin polymerization, subsequently leading to reduced neutrophil migration (Krishnamoorthy et al., 2010). These effects were also observed *in vivo*, as treatment with DHA resulted in reduced rolling, adhesion, and adhesion efficiency in the cremaster muscle microcirculation. Similar effects were observed in the peritonitis CLP model treated with omega 3 lipid emulsions (Korner et al., 2018). Subsequently, we evaluated whether DHA modulates the expression of key adhesion molecules mediating leukocyte-endothelial interactions. We observed that DHA treatment reduced the expression of CXCR4, PSGL-1, and CD11a on isolated neutrophils. DHA pretreatment reduced the expression of other adhesion molecules, such as CD11b and CD47, both of which are involved in neutrophil recruitment (Jensen et al., 2020).

Additionally, by downregulating chemokine receptors, such as CXCR4, on neutrophils, the ability of these cells to respond to chemotactic signals is impaired, leading to further reductions in neutrophil recruitment to sites of inflammation (De Filippo and Rankin, 2018). Sepsis is defined as life-threatening organ dysfunction caused by a dysregulated host response to infection, characterized by profound circulatory, cellular, and metabolic abnormalities that significantly elevate the risk of mortality (Singer et al., 2016). Sepsis disrupts the integrity of the blood-brain barrier (BBB), leading to increased permeability. This allows pro-inflammatory cytokines and neurotoxic substances to enter the brain, exacerbating neuronal injury and contributing to cognitive impairments. The inflammatory response can also impair the cerebral microcirculation, reducing blood perfusion and oxygen delivery to brain tissues (Gao and Hernandes, 2021; Manabe and Heneka, 2022). DHA exerts its anti-inflammatory effects through both direct and indirect mechanisms. Directly, DHA interacts with nuclear receptors such as peroxisome proliferator-activated receptor (PPAR). Inhibiting the expression of pro-inflammatory cytokines and adhesion molecules, reducing leukocyte adhesion and rolling in the vasculature (Calder, 2017). Similarly, nuclear factor erythroid 2–related factor 2 (NRF2) activation by DHA promotes the expression of antioxidant response elements, which further counteract inflammation (Zgorzynska et al., 2021). Interestingly, DHA can also influence membrane dynamics and signaling by modifying the lipid composition of cell membranes (Torres et al., 2021; Li et al., 2022). By displacing arachidonic acid (AA) from cellular membrane phospholipids, DHA decreases the availability of substrates required for the synthesis of pro-inflammatory eicosanoids, such as prostaglandins and leukotrienes, thereby contributing to a lipid mediator class switching toward the production of pro-resolving mediators (SPM) (Buckley et al., 2014; Hansen et al., 2018), such as resolvins and protectins, which have potent anti-inflammatory and pro-resolving effects. These SPM resolve inflammation, rather than merely suppressing it, by limiting neutrophil infiltration, promoting macrophage clearance, and restoring tissue homeostasis (Ghodsi et al., 2024; Sun et al., 2024).

To understand the effects of a single dose of DHA in sepsis, we used the established CLP model of murine polymicrobial sepsis, which closely resembles bacterial peritonitis-induced sepsis in humans and is currently considered the gold standard in sepsis research (Dejager et al., 2011). The bioavailability of a single dose of DHA has been examined in a comprehensive review, comparing different chemical forms, which demonstrates that non-esterified fatty acids exhibit the highest absorption efficiency (Alijani et al., 2025). Several single-dose human studies using various omega-3 formulations and compositions reported substantial increases in circulating biomarkers (Alijani et al., 2025). Despite the limitation of a single dose, randomized controlled trials have shown that plasma total lipid EPA + DHA levels increase over 12 h with both formulations (Fisk et al., 2021) and over 24 h with a DHA self-microemulsifying delivery system (SMEDS) (West et al., 2018). Some studies suggested that consuming at least 2,400 mg/day of omega-3 fatty acids (EPA + DHA) for at least 4.5 weeks may be adequate in healthy individuals (Fernandez-Lazaro et al., 2024). A dose-response meta-analysis further indicated that the optimal combined intake for blood pressure reduction is likely between 2 and 3 g per day (Zhang et al., 2022). Most of these studies exhibit bias toward the specific health outcomes they were designed to evaluate, limiting the generalizability of conclusions about optimal dosing.

Nevertheless, significant beneficial effects of DHA or omega-3 supplementation have been reported across several conditions, including depression (Wu S. K. et al., 2024), prostate cancer (Aronson et al., 2025), and age-related macular degeneration (Xue et al., 2025), among others. In our study, DHA administration improved survival in septic mice, effectively restoring body temperature and markedly improving sickness behaviors, as evidenced by an amelioration in clinical scores. Treatment with n-3 PUFAs enhance survival rates following CLP-induced sepsis (Chen et al., 2022; Chen et al., 2023). Several studies have demonstrated the improvement of phagocytic capacity of DHA-treated immune cells by activating Src/Syk signaling-dependent phagocytosis or regulating other pathways such as cAMP, PKA, and STAT3 (Chiang et al., 2017). Inhibition of Src pathways is strongly correlated with reduced cellular infiltration and bacterial growth in an experimental sepsis model (Cunha et al., 2025). Accordingly, these pathways might contribute to the lower bacterial load observed in the peritoneal exudate of CLP mice pretreated with DHA.

DHA treatment also significantly reduced the total number and percentage of transmigrated neutrophils into the peritoneal cavity. In an earlier report, DHA administration inhibited neutrophil infiltration in a colitis model (Ariturk et al., 2024). Notably, Resolvin D3 also exhibits anti-inflammatory effects by reducing neutrophil transmigration in a murine peritonitis model (Dalli et al., 2013), with comparable effects observed for maresin 1 (Sun et al., 2023) and protectin D1 (Wu et al., 2021).

We also evaluated whether DHA treatment would modulate one of the hallmark characteristics of sepsis, the massive cytokine release, assessing its effects locally, systemically, and within brain tissue. In our model, DHA treatment reduced key pro-inflammatory cytokines and chemokines, consistent with its known mechanism of action, which involves inhibition of major pro-inflammatory transcription factors such as NF-κB and AP-1 (Zgorzynska et al., 2021). Plasma BDNF has been considered a more reliable biomarker than the erythrocyte omega-3 index for assessing omega-3 fatty acid enrichment in the brain (Sugasini et al., 2020). A bidirectional transfer of BDNF between the brain and plasma has been demonstrated, with up to 80% of plasma BDNF potentially derived from the brain. There is compelling evidence that DHA increases BDNF expression in the brain, possibly via Akt or GPR40 activation (Sugasini et al., 2020). In our study, DHA treatment resulted in a significant increase in plasma BDNF levels in both the sham and CLP groups.

We quantified resolvin D1 and D2 in plasma and brain tissue, observing increases in all DHA-treated groups compared with the SHAM and CLP saline groups. That evidence proves DHA is indeed metabolized into the SPM resolvin D.

DHA has been shown to enhance endothelial function, a critical factor in preserving vascular health and tissue perfusion (Zehr and Walker, 2018). Even in the absence of hypoperfusion, sepsis is frequently associated with elevated lactate levels due to multiple non-hypoxic mechanisms, as impaired lactate clearance (Zhang et al., 2024). Mitochondrial dysfunction (Wu Z. et al., 2024), enhanced adrenergic stimulation, and metabolic reprogramming can increase glycolytic flux and lactate production despite adequate oxygen delivery (Zhang et al., 2025). DHA influences vascular tone by modulating the production of nitric oxide and endothelin-1 in endothelial cells, thereby promoting a balance between vascular relaxation and constriction (Yamagata, 2017). DHA improved perfusion in CLP mice and reduced lactate levels, indicating enhanced cerebral perfusion.

Our study has limitations that should be acknowledged. First, although multiple DHA doses could be explored in future studies to establish a dose–response relationship, a single dose was sufficient to elicit measurable effects. Nevertheless, a single administration limits translational applications. The observed increase in resolvin levels was interpreted as an indirect indicator of DHA incorporation; however, we did not block the resolvin pathway or its receptor, which limits the mechanistic interpretation regarding the specific role of resolvins. Another limitation is the absence of female and aged animals, which may exhibit distinct responses due to hormonal influences and age-related alterations in inflammation that should be addressed in future studies. Finally, the use of total brain tissue precluded the identification of specific cellular sources of cytokine production within the central nervous system. Despite these limitations, we genuinely believe we have brought new insights into DHA’s actions in diseases characterized by overshooting inflammation, such as sepsis.

Finally, we evaluated leukocyte–endothelial interactions in the brain and observed reduced leukocyte rolling and adhesion in the microcirculation of DHA-treated septic mice. This reduction is particularly significant in sepsis, where excessive leukocyte recruitment contributes to tissue damage and organ dysfunction. DHA exerts anti-inflammatory effects by modulating the expression of adhesion molecules on endothelial cells. By downregulating pro-inflammatory cytokines and adhesion molecules such as E-selectin and VCAM-1, DHA reduces leukocyte adhesion and transmigration across the endothelium, mitigating inflammation-induced vascular damage (Wang et al., 2011; Yang et al., 2013). Taken together, this study provides evidence that ultrapure DHA, an omega-3 fatty acid, exerts substantial anti-inflammatory and pro-resolving effects by modulating leukocyte-endothelial interactions, reducing leukocyte adhesion and rolling, and improving microcirculatory function in localized inflammation and systemic sepsis. The observed reductions in mortality, cytokine levels, and improvements in perfusion in the CLP sepsis model highlight DHA’s potential to mitigate key aspects of sepsis pathology. DHA’s ability to stabilize vascular integrity and enhance perfusion could be an interesting adjunctive therapy for sepsis, targeting vascular dysfunction and limiting organ damage. Future studies should further investigate DHA’s mechanisms and clinical applicability in inflammatory conditions such as sepsis.

## Data Availability

The raw data supporting the conclusions of this article will be made available by the authors, without undue reservation.
