# Correction: Omega 3 fatty acid docosahexaenoic acid (DHA) mitigates inflammatory responses in experimental sepsis

**DOI:** 10.3389/fphar.2026.1782326

**Published:** 2026-01-22

**Authors:** Bianca Portugal Tavares de Moraes, Isabelle Moraes-de-Souza, Gabrielle Lacerda de Souza Gomes-Reis, Marina Ferreira-Costa, Carolina Medina Coeli da Cunha, Matheus Augusto Patricio De Almeida, Vanessa Estato, Kauê Francisco Corrêa Souza e Souza, Francisco da Silva dos Santos, Maria Alice dos Santos Mascarenhas Brito, Patrícia Novaes Soares, Wilza Arantes Ferreira Peres, Roland Immler, Matteo Napoli, Patrícia Torres Bozza, Hugo Caire de Castro-Faria-Neto, Markus Sperandio, Adriana Ribeiro Silva, Cassiano Felippe Gonçalves-de-Albuquerque

**Affiliations:** 1 Immunopharmacology Laboratory, Federal University of State of Rio de Janeiro, Rio de Janeiro, Brazil; 2 Post-Graduation Program in Molecular and Celular Biology, Federal University of State of Rio de Janeiro, Rio de Janeiro, Brazil; 3 Immunopharmacology Laboratory, Oswaldo Cruz Institute, Fiocruz, Rio de Janeiro, Brazil; 4 Post-Graduation Program in Molecular and Celular Biology, Oswaldo Cruz Institute, Fiocruz, Rio de Janeiro, Brazil; 5 Post-Graduation Program in Neuroscience, Fluminense Federal University, Rio de Janeiro, Brazil; 6 Department of Nutrition and Dietetics - Josué de Castro, Nutrition Institute, Federal University of Rio de Janeiro, Rio de Janeiro, Brazil; 7 Institute of Cardiovascular Physiology and Pathophysiology, Biomedical Center, Ludwig-Maximilians-University Munich, Munich, Germany

**Keywords:** docosahexaenoic acid, omega 3, microcirculation, neuroinflammation, intravital microscopy, neutrophils, clp

There was a mistake in Figure 7 as published. In Figure 7A, the panels are incorrectly labelled. ‘SHAM + SAL’ was incorrectly captured as ‘SHAM + LNC_BL_’; ‘SHAM + DHA’ was incorrectly captured as ‘SHAM + LNC_DHA_’; ‘CLP + SAL’ was incorrectly captured as ‘CLP + LNC_BL_’; and ‘CLP + DHA’ was incorrectly captured as ‘CLP + LNC_DHA_’. The corrected Figure 7 appears below:

**FIGURE 7:**
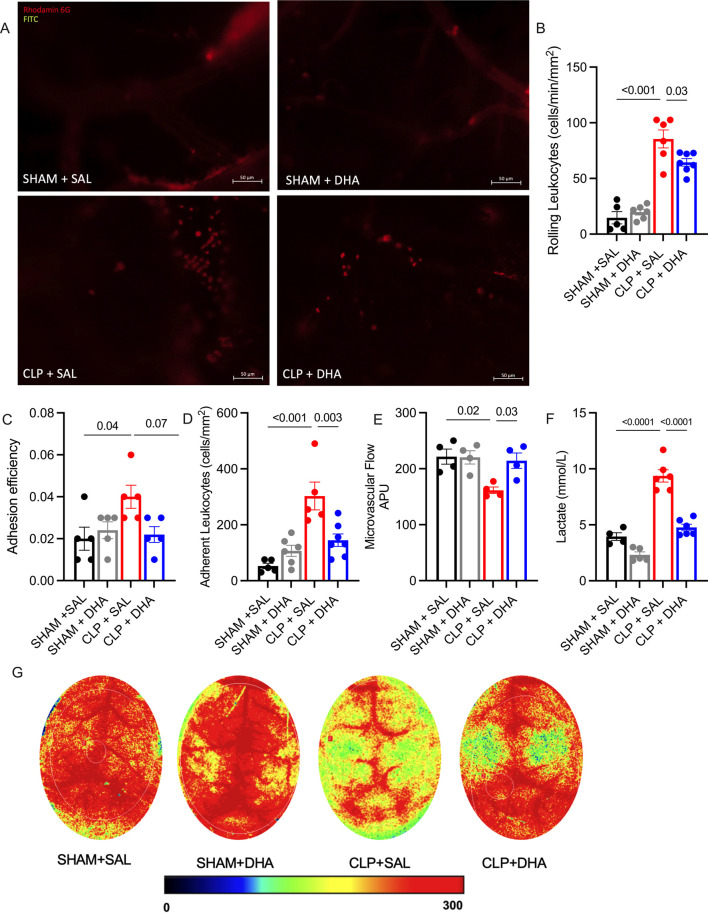
DHA improved microcirculation and perfusion in septic mice. Intravital microscopy analysis of cerebral microcirculation in animals induced with sepsis by CLP and treated with DHA 200 mg/kg. **(A)** Representative image of blood vessels under intravital microscopy for each group. **(B)** Rolling cells fraction per minute per mm^2^, **(C)** Adherent cells per mm^2^, **(D)** Leukocyte adhesion efficiency to the endothelium, **(E)** Lactate levels in plasma. **(F)** Blood flow intensity assessed by laser speckle imaging, expressed in Arbitrary Perfusion, and **(G)** Representative images of perfusion under laser speckle for each group 24 h after sepsis induction by CLP. *p < 0.05, ****p < 0.001, as determined by one-way ANOVA with Bonferroni multiple comparison test. N = 4-6. Data were calculated from at least 8 postcapillary venules from 4 to 5 mice.

The original article has been updated.

