# Supplementary material for: Omega 3 fatty acid docosahexaenoic acid (DHA) mitigates inflammatory responses in experimental sepsis
Source: Front Pharmacol. 2025 Nov 28;16:1708348. doi: 10.3389/fphar.2025.1708348 (PMC12698574; doi:10.3389/fphar.2025.1708348)
Supplement: Supplementary file 3 [file Table1.docx]

Supplementary Material


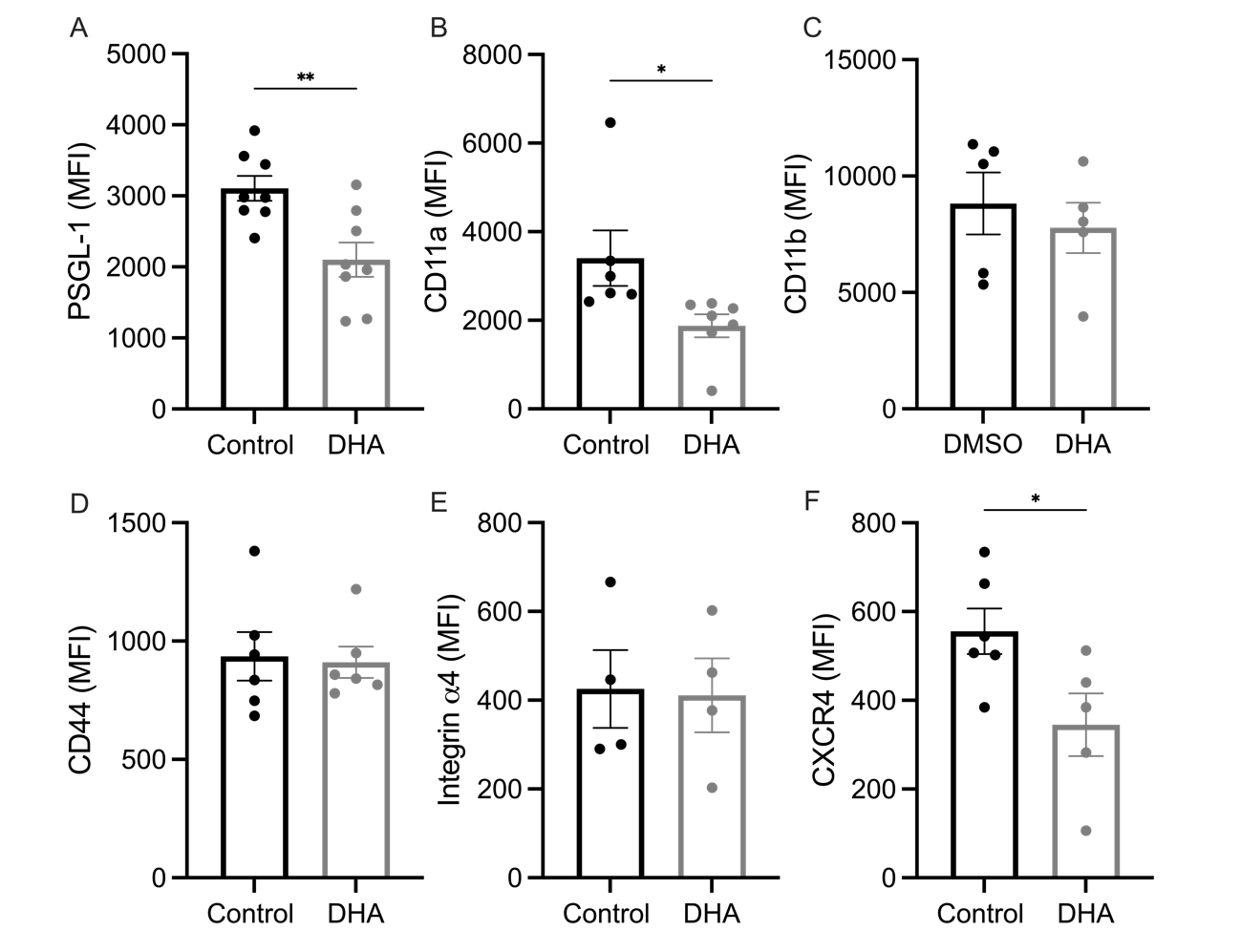


**Supplementary Fig. 1** Treatment with reduced the expression of adhesion molecules in neutrophils. Analysis was conducted via flow cytometry on neutrophils isolated from the bone marrow of mice and treated for 3 hours with DHA 100 μM, along with control. A. CXCR4, B. PSGL-1, C. CD11a, D. CD44, E. Integrin α4. The values were expressed as “mean fluorescence intensity”. Values are means with SD presented with vertical bars; * p < 0.05, *** p < 0.005, as determined by two-tailed unpaired Student’s t-tests. n=4-6

| **Antibody** | **Conjugate** | **Host** | **Isotype** | **Clone** | **Company** |
| --- | --- | --- | --- | --- | --- |
| CD11a (LFA-1) | PE | Rat | IgG2a k | M17/4 | Biolegend |
| CD11b (Mac-1) | PE | Rat | IgG2b k | M1/70 | Biolegend |
| CD45 | PerCP-Cy5.5 | Rat | IgG2b k | 30F-11 | Biolegend |
| CD184 (CXCR4) | FITC | Rat | IgG2b | 2b11 | Pharmigen |
| Ly6G | PB | Rat | IgG2a k | 1A8 | Biolegend |
| CD162 (PSGL-1) | PerCP-Cy5.5 | Rat | IgG1 | 2PH1 | Pharmigen |
| CD49d | FITC | Rat | IgG2b k | 30-H12 | Biolegend |
| CD44 | BV570 | Rat | IgG2b k | IM7 | Biolegend |
| IgG1 | PerCP-Cy5.5 | Rat | IgG1 k | RTK2071 | BioLegend |
| IgG2a k | PB | Rat | IgG2a k | RTK2758 | Biolegend |
| IgG2b | FITC | Rat | IgG2b |  | Santa Cruz |
| IgG2b k | BV 570 | Rat | IgG2bk | RTK4530 | Biolegend |
| IgG2b k | FITC | Rat | IgG2b k | RTK4530 | Biolegend |

**Supplementary Table 1.** List of antibodies used in flow cytometry experiments.

|  | **Mice**  **(n)** | **Venules**  **(n)** | **Diameter**  **(µm)** | **Centerline velocity**  **(µm s–1)** | **Wall shear**  **rate (s–1)** | **WBC**  **(µl–1)** |
| --- | --- | --- | --- | --- | --- | --- |
| Control | 5 | 30 | 43,6±1,2 | 890±90 | 620±30 | 1800±160 |
| DHA | 6 | 38 | 39,8±2,6 | 880±140 | 700±40 | 1700±400 |

**Supplementary Table 2.** Microvascular and hemodynamic parameters of intravital microscopy experiments in of the mouse cremaster muscle (Figure 2).
